# Histology-validated quantitative MRI characterization of collagen-rich Purkinje fiber-associated pathways in the *ex vivo* porcine heart

**DOI:** 10.1186/s41747-026-00793-0

**Published:** 2026-09-15

**Authors:** Yi Li, Victor Casula, Manu Pradeep, Timo Liimatainen

**Affiliations:** 1 Research Unit of Health Sciences and Technology, University of Oulu, Oulu, Finland; 2 Medical Research Center Oulu, Oulu University Hospital, University of Oulu, Oulu, Finland; 3 Department of Radiology, Oulu University Hospital, Oulu, Finland

**Keywords:** Collagen (magnetic resonance imaging), Heart conduction system, Histology, Purkinje fibers

## Abstract

**Objective:**

Collagen-rich Purkinje fiber-associated (PF-associated) pathways within the subendocardial layer are small, compositionally distinct structures that are challenging to characterize noninvasively. This study aimed to evaluate whether quantitative magnetic resonance imaging (qMRI) can characterize collagen-rich PF-associated pathways using histology as a structural ground truth.

**Materials and methods:**

*Ex vivo* porcine left ventricular specimens were imaged at 3 T using relaxation-based mapping techniques, including *T*_RAFF_, *T*_1ρ_, *T*_2_ and *T*_1_ mapping. Corresponding Masson’s trichrome sections were processed into optical density maps to segment myocardium, collagen and PF-associated regions. Histology-defined regions of interest were used to identify collagen-enriched PF-associated pathways and remote myocardium. Relaxation time contrast was quantified using relative relaxation time difference (RRTD). Statistical comparisons were performed using paired *t*-tests with Benjamini–Hochberg correction.

**Results:**

Histology confirmed collagen-enriched PF-associated pathways within subendocardial tissue and false tendons. Optical density-based segmentation reliably differentiated tissue components with low inter-section variability. Collagen-rich PF-associated regions showed significantly higher *T*_RAFF_, *T*_1ρ_, *T*_2_, and *T*_1_ values compared with remote myocardium. RRTD analysis indicated that *T*_RAFF_ and *T*_1ρ_ provided the strongest relative contrast among the evaluated parameters.

**Conclusion:**

Histology-validated qMRI enables characterization of collagen-rich PF-associated pathways in the *ex vivo* porcine heart. These findings demonstrate the sensitivity of relaxation-based MRI techniques to collagen-rich PF-associated pathways and support further investigation of cardiac conduction system imaging.

**Key points:**

- ***Question*** Noninvasive MRI characterization of collagen-rich PF-associated pathways remains challenging because these small conduction structures are difficult to identify.
- ***Findings*** Histology-referenced MRI differentiated collagen-rich PF-associated pathways from those of the remote myocardium, with rotating-frame relaxation methods providing the strongest contrast.
- ***Relevance statement*** qMRI relaxation mapping enables histology-validated characterization of collagen-rich PF-associated pathways, supporting the development of noninvasive imaging approaches for studying cardiac conduction system microstructure.

**Graphical Abstract:**

**Figure:**
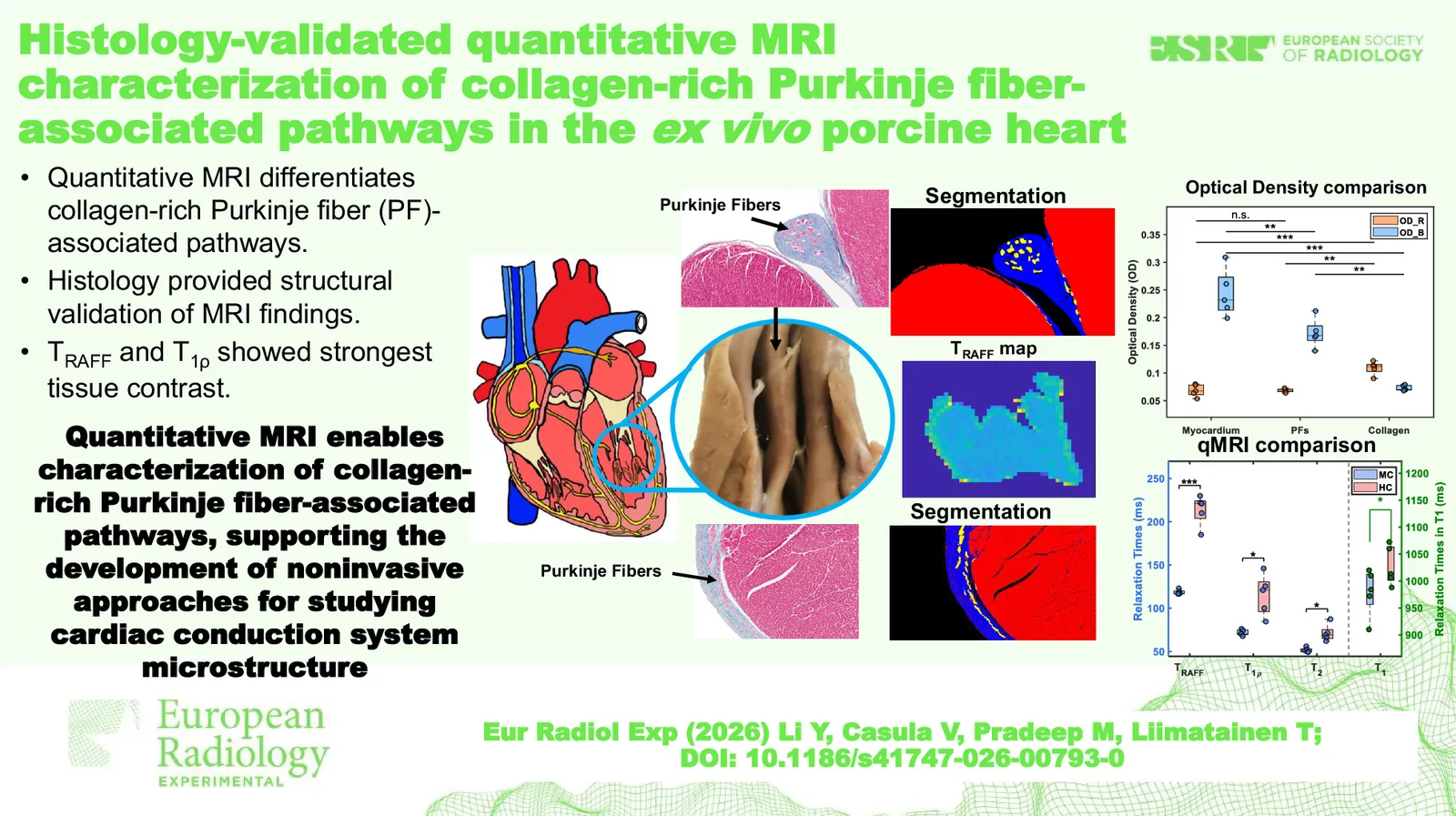

## Introduction

The cardiac conduction system (CCS) consists of specialized myocytes that initiate and propagate electrical impulses to coordinate ventricular activation [1]. Its terminal component, the Purkinje fibers (PFs), forms a high-velocity conduction network that ensures near-synchronous ventricular contraction [2]. Structurally, PFs are embedded within a highly organized extracellular matrix (ECM) composed of subendocardial connective layers and false tendons (FTs) [3]. FTs are intracavitary fibromuscular bands predominantly composed of dense collagen bundles that surround and support PF bundles, providing structural stabilization and defining their anatomical trajectories [4, 5]. Because PFs are thin and sparsely distributed within the ventricular myocardium, they cannot be directly visualized using current *in vivo* magnetic resonance imaging (MRI) approaches. Instead, MRI relaxation contrast primarily reflects the composition and microstructural properties of surrounding collagen-rich ECM associated with PF pathways.

Masson’s trichrome staining enables clear visualization of collagen (blue) and myocardium (red), thereby outlining the fibrous scaffolds through which PFs course [6]. Although PFs lack a specific histochemical marker, their distribution within collagen-rich substrates, particularly the subendocardial layer and fibrous trabeculae, provides consistent anatomical landmarks for delineating their spatial organization [7]. Consequently, histology-derived ground truth serves as a biological reference for evaluating whether collagen-enriched PF regions generate detectable imaging signatures in quantitative MRI (qMRI).

qMRI enables non-destructive characterization of myocardial microstructure. Conventional *T*_1_ and *T*_2_ mapping reflect global alterations in water content and fibrosis but have limited specificity for matrix-level interactions [8]. Rotating-frame relaxation methods, including longitudinal rotating frame relaxation time (*T*_1ρ_) and relaxation along a fictitious field (*T*_RAFF_), are sensitive to slow molecular motions and macromolecular environments, improving contrast in collagen-rich or structurally specialized myocardial regions [9, 10]. *T*_RAFF_ is generated using amplitude- and frequency-modulated RAFF pulses, which improve clinical feasibility by reducing RF power deposition while demonstrating increased relaxation times in fibrotic myocardium [9, 11, 12].

Previous studies have shown that *T*_1ρ_ and *T*_RAFF_ can delineate specialized conduction structures such as the sinoatrial node and components of the atrioventricular conduction axis, indicating sensitivity to regions with distinctive cellular organization and dense ECM [13, 14]. The pig heart is an appropriate model for CCS imaging because its ventricular geometry, myocardial fiber architecture, and Purkinje system organization closely resemble those of the human heart [3]. Although pigs exhibit slightly more extensive subendocardial arborization, overall PF morphology and ECM-associated pathways are well conserved, supporting their use in *ex vivo* CCS studies [15].

Previous *ex vivo* work using magnetization transfer (MT) imaging demonstrated the feasibility of visualizing free-running PF structures at preclinical ultra-high field strength (9.4 T) [16]. In contrast, the present study focuses on quantitative relaxation mapping techniques, acquired at clinically compatible 3-T field strength. Unlike MT contrast, which depends strongly on acquisition parameters and field strength, rotating-frame relaxation techniques provide quantitative measures sensitive to water-macromolecule interactions within PF-associated ECM pathways.

Despite recent advances in qMRI and MT-based imaging approaches, characterization of PF-associated ECM pathways remains challenging due to their complex microstructural organization and limited intrinsic contrast relative to surrounding myocardium [16]. This limitation highlights the need for imaging strategies capable of characterizing PF-associated ECM pathways with sufficient contrast and spatial specificity, while maintaining translational potential for *in vivo* application. Therefore, this study aimed to evaluate whether rotating-frame relaxation time mapping, compared with conventional *T*_1_ and *T*_2_ mapping, can characterize collagen-rich PF-associated ECM pathways using histology-derived optical density (OD) maps as structural reference.

## Materials and methods

### Samples preparation

**Fig. 1:**
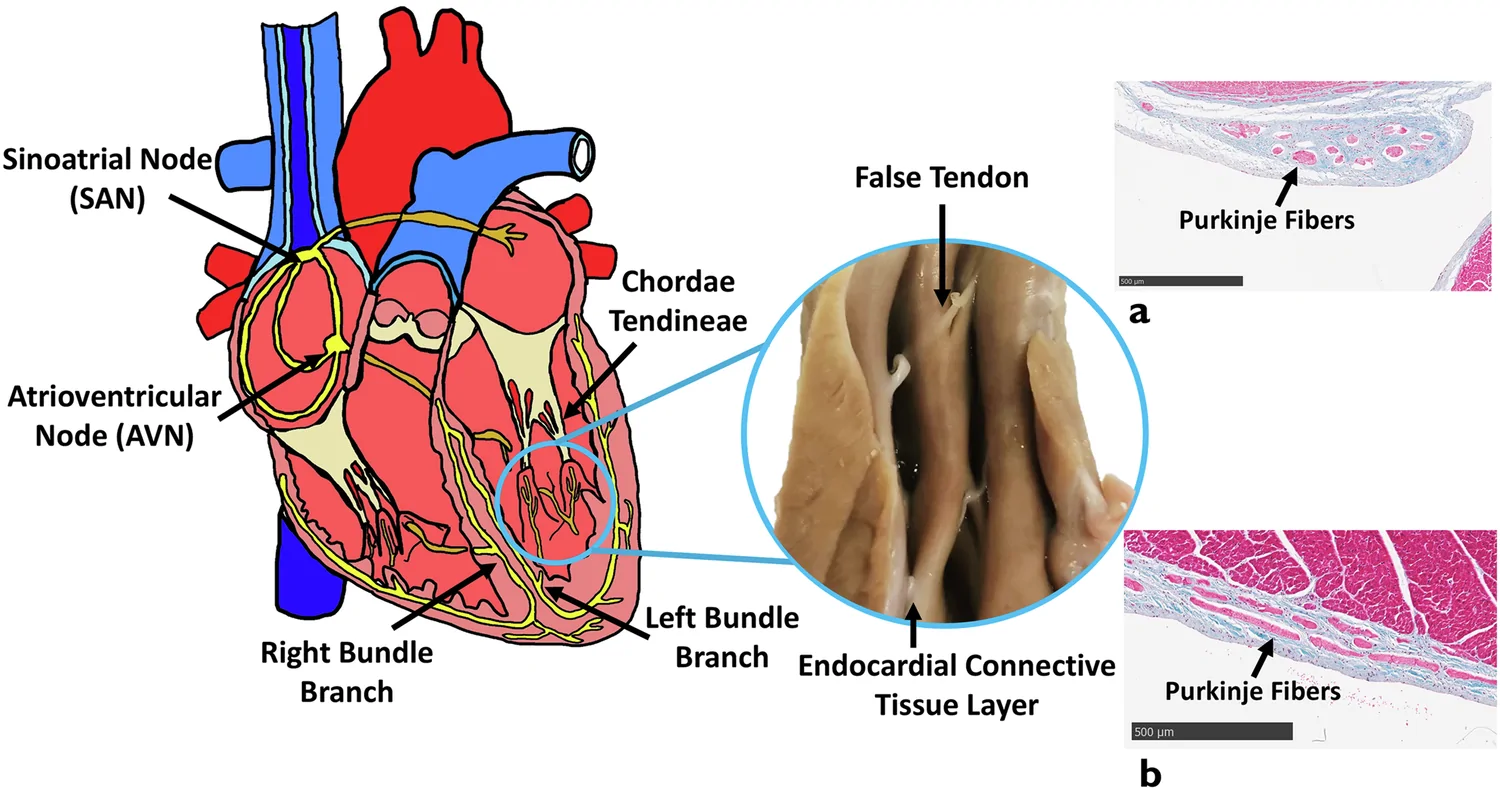
Schematic illustration of the anatomical organization of the CCS in the left ventricle. The enlarged view indicates the approximate location of the subendocardial myocardium from which tissue samples were collected. This region includes the subendocardial connective tissue layer and a representative FT spanning the ventricular cavity. PFs are depicted (**a**) within the cross section of FT and (**b**) along the subendocardial surface, demonstrating their close spatial relationship with the surrounding connective tissue and adjacent working myocardium. This diagram provides anatomical context for the subsequent MRI and histological analyses

Five hearts from Finnish Landrace pigs were obtained from a local abattoir immediately after routine slaughter for food production. The hearts were vacuum-packed and transferred to the laboratory on ice. Specimens were stored at approximately 3 °C for less than 24 h before dissection. Five *ex vivo* tissue blocks were dissected from the apical region of the left ventricular wall. After opening the hearts, residual blood was removed by rinsing with cold phosphate-buffered saline. Tissue blocks containing the endocardial surface, subendocardial layer, adjacent working myocardium, and visible FTs were carefully trimmed while preserving the anatomical orientation of Purkinje fiber-associated regions (Fig. 1). Before MRI measurements, each tissue block was photographed to document orientation and transferred into a sealed plastic imaging container. The specimens were fully immersed in perfluoropolyether oil (Fomblin, Solvay Solexis), which provides a proton-signal-free background and minimizes susceptibility differences between the specimen and surrounding medium. Due to the complexity of the *ex vivo* imaging and histological workflow, the present work was designed as an exploratory feasibility study.

### MRI protocol

The porcine heart specimens were imaged on a 3-T MRI scanner (MAGNETOM Vida, Siemens Healthineers) using an 18-channel knee coil. Each specimen, immersed in perfluoropolyether oil, was positioned in a sealed plastic container and placed at the center of the coil to ensure optimal signal uniformity. Imaging was performed under stable room-temperature conditions (approximately 20–22 °C) within the MRI environment to minimize tissue deformation and experimental variability, and care was taken to avoid air bubbles within the container. No noticeable temperature fluctuations were observed during the imaging sessions. During MRI, multiple slices (typically 15–25 per specimen) were acquired perpendicular to the trajectory of FTs to ensure full coverage of the tissue block. *T*_RAFF_, *T*_1ρ_, *T*_2_, and *T*_1_ relaxation times were measured with an in-plane resolution of 0.6 × 0.6 mm², acquisition matrix 156 × 192, field of view (FOV) of 90 × 110 mm², and slice thickness 1.5 mm. The readout bandwidths were 260 Hz/pixel for *T*_RAFF_ and *T*_1ρ_, 299 Hz/pixel for *T*_2_, and 248 Hz/pixel for T_1_. The total MRI acquisition time for each specimen was approximately 2–3 h across all imaging sequences.

For RAFF2, the maximum RF pulse amplitude was 500 Hz with a pulse duration of 2.82 ms. Rank-2 RAFF pulse trains of 0, 8, 16, 24, and 32 pulses were applied to generate RAFF-weighted images. The corresponding relaxation time constant is referred to as *T*_RAFF_. *T*_1ρ_ measurements were performed using a continuous-wave (CW) spin-lock pulse with an amplitude of 500 Hz and five spin-lock times (TSLs) ranging from 0 to 60 ms. The spin-lock preparation was based on previously described refocused spin-lock approaches designed to improve robustness against B0 and B1 inhomogeneity effects [17]. The RAFF2 and *T*_1ρ_ preparation modules were followed by a spoiled gradient-echo readout (repetition time 3 s, echo time 4.86 ms, flip angle 25°). For *T*_2_ mapping, multi-echo acquisitions were performed with echo times ranging from 10 to 50 ms in 10 ms increments (repetition time 3 s). T_1_ relaxation times were obtained using an inversion recovery preparation with six inversion times ranging from 200 to 3,900 ms. Each inversion recovery module was followed by a spoiled gradient-echo readout (repetition time 4.09 s, echo time 9.6 ms).

### Histology

Following MRI acquisition, all samples were fixed in 10% neutral buffered formalin for 24–48 h, and subsequently dehydrated through a graded ethanol series, cleared in xylene, and embedded in paraffin. Serial histology sections (5 µm thickness) were cut longitudinally using a microtome and mounted onto glass slides. Several consecutive sections from each tissue sample were selected for staining to ensure coverage of both the endocardial surface and any visible FTs.

Histological staining was performed using standard Masson’s trichrome staining protocol to differentiate myocardium, collagen, and PF-associated regions. All slides were stained manually by the same operator to maintain procedural consistency across samples. After staining, sections were digitized using the NanoZoomer S60 whole-slide scanner (C1321001) under standardized illumination and magnification settings. Digital images were reviewed using NDP.view2 software (U12388-01).

### Histological image analysis

**Fig. 2:**
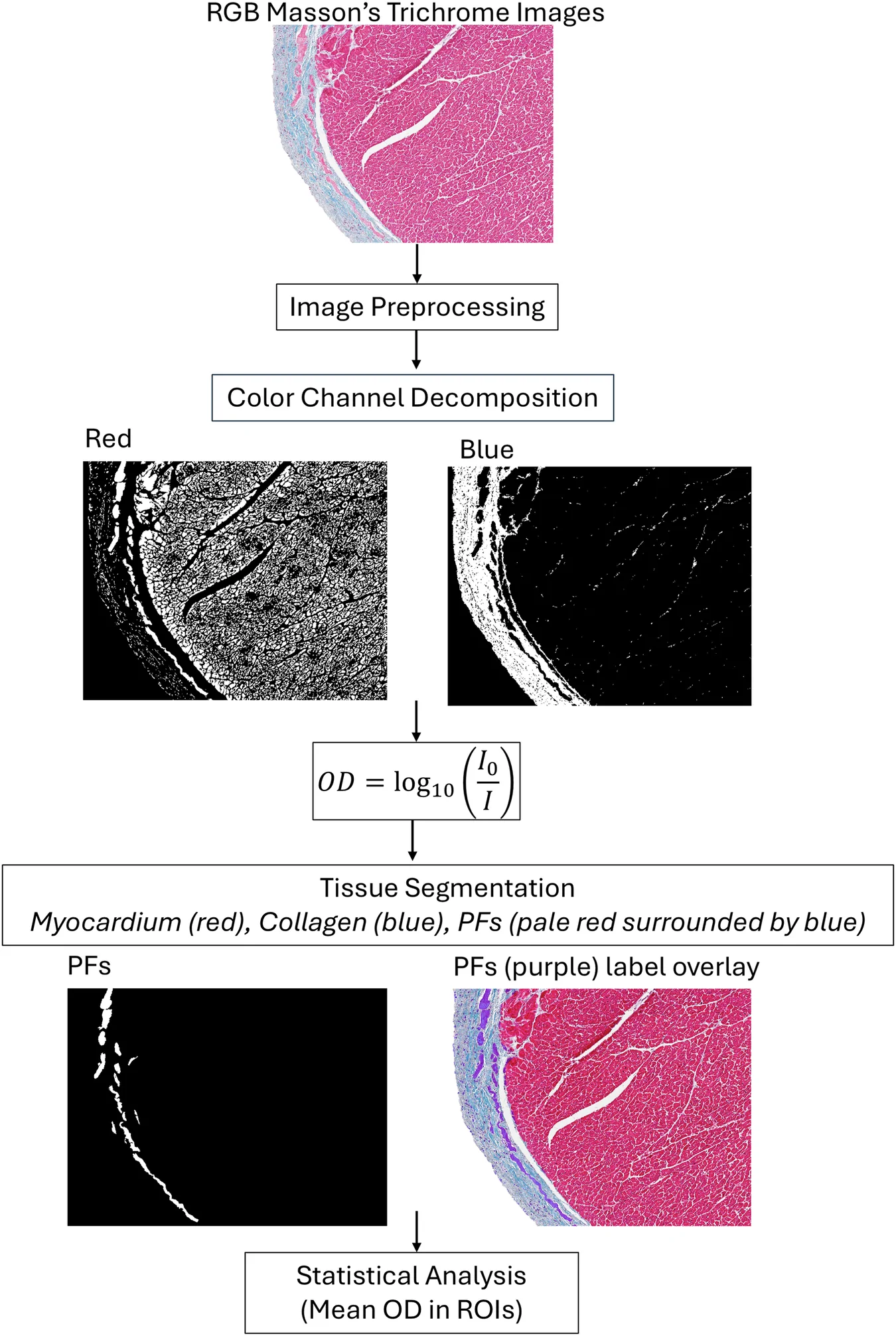
Schematic of the histology analysis pipeline from RGB images to quantitative OD comparison among tissue types. RGB images were decomposed into red and blue channels, converted into OD maps, and segmented to identify myocardium, PFs, and collagen. Mean OD values were statistically compared across tissue types

Quantitative histological analysis was performed by decomposing Masson’s trichrome RGB images into red and blue channels, converting them into OD maps, and segmenting tissue types based on characteristic staining patterns (Fig. 2). Myocardium was identified from regions with predominant red channel intensity, collagen from regions with predominant blue channel intensity, and PF-associated regions from red-stained myocardial signal embedded within collagen-rich strands. Segmentation thresholds were applied consistently across all sections under identical processing conditions. OD was computed using the standard formula: OD = log_10_ (*I*_0_/*I*), where *I* is the measured pixel intensity and *I*_0_ is the background reference intensity. OD_R and OD_B refer to the optical densities derived from the red and blue channels, respectively, corresponding to myocardium and collagen staining in Masson’s trichrome sections.

Mean OD_R and OD_B values were first calculated for each tissue region of interest (ROI) within each histological section, including myocardium, PF-associated regions, and collagen. For each heart, values from two anatomically corresponding sections were averaged to obtain one heart-level value per tissue type, and these values were used for the primary statistical analysis. Given the exploratory, feasibility-oriented nature of the study and the limited sample size, paired statistical comparisons were performed between myocardium and PF-associated regions, and between myocardium and collagen, for both OD_R and OD_B using paired *t*-tests with Benjamini–Hochberg correction for multiple comparisons. Effect sizes and absolute differences with confidence intervals were additionally reported for the main comparisons.

To assess section-to-section reproducibility, five consecutive sections from one representative sample were processed and analyzed under identical staining and segmentation conditions. This analysis provided an internal consistency evaluation of the staining workflow and semi-automated tissue classification procedure.

### MRI data analysis

Relaxation time maps were generated using pixel-wise fitting of the signal intensity images with the Aedes software package (http://aedes.uef.fi/) implemented in MATLAB (R2024a, MathWorks Inc.). RAFF relaxation time maps were calculated from the weighted images by fitting the corresponding signal model, $S(t)={S}_{0}{e}^{-\frac{t}{{T}_{{{\rm{RAFF}}}}}}-{SS}(1-{e}^{-\frac{t}{{T}_{{{\rm{RAFF}}}}}})$, where the steady-state term (SS) represents the fraction of signal intensity relative to the initial magnetization ${S}_{0}$ after RAFF weighting [11]. *T*_1ρ_, *T*_2_, and *T*_1_ maps were fitted using standard mono-exponential signal decay models.

Histological sections and MRI slices were matched based on slice-level anatomical correspondence using visible structural landmarks, including the endocardial surface, subendocardial layer, FTs, and surrounding myocardial geometry. No formal image co-registration between MRI and histology was performed because histological processing introduced tissue deformation that limited precise spatial alignment between modalities. Instead, histological sections and MRI slices were matched based on slice-level anatomical correspondence using visible structural landmarks, including the endocardial surface, subendocardial layer, FTs, and surrounding myocardial geometry. Histological OD maps were first used to identify myocardium, collagen-rich regions, and PF-associated regions, and MRI ROIs were subsequently defined with reference to the corresponding histological findings and anatomical landmarks.

Collagen-rich regions, including FTs and subendocardial connective layers, were identified exclusively based on histological segmentation of Masson’s trichrome sections. Regions of interest (ROIs) corresponding to histology-defined collagen-rich PF-associated areas were labeled as high collagen (HC), whereas ROIs in remote myocardium were labeled as myocardial control (MC). HC ROIs were manually delineated by a single observer using the histology-referenced anatomical correspondence framework described above. To reduce potential bias toward artificially increased contrast, HC ROIs were conservatively drawn to include the broader anatomically corresponding collagen-rich PF-associated region rather than only visually high-intensity pixels. ROI size varied according to the anatomical extent and geometry of the PF-associated structures in each specimen. MC ROIs were positioned in remote myocardial regions away from visible collagen-rich structures and transitional zones. For each relaxation parameter (*T*_RAFF_, *T*_1ρ_, *T*_2_, and *T*_1_), relaxation times were averaged separately within HC and MC ROIs. The contrast in the relaxation time maps was quantified using the relative relaxation time difference (RRTD), defined as: RRTD = 2[*T*(HC) − *T*(MC)]/[*T*(HC) + *T*(MC)], where *T* represents the mean relaxation time within the corresponding ROI.

Data are presented as mean ± standard deviation. No formal a priori sample size or power calculation was performed, as this study was designed as an exploratory *ex vivo* feasibility study. Paired comparisons between HC and MC regions were assessed using paired *t*-tests. Comparisons of RRTD values among qMRI parameters were performed using repeated-measures one-way ANOVA followed by post hoc pairwise comparisons with Benjamini–Hochberg correction for multiple testing. Because formal assessment of normality is inherently limited with *n* = 5, statistical findings were interpreted cautiously and complemented by effect sizes, absolute differences, confidence intervals, and consistency of directional differences across samples. A corrected *p*-value < 0.05 was considered statistically significant.

## Results

**Fig. 3:**
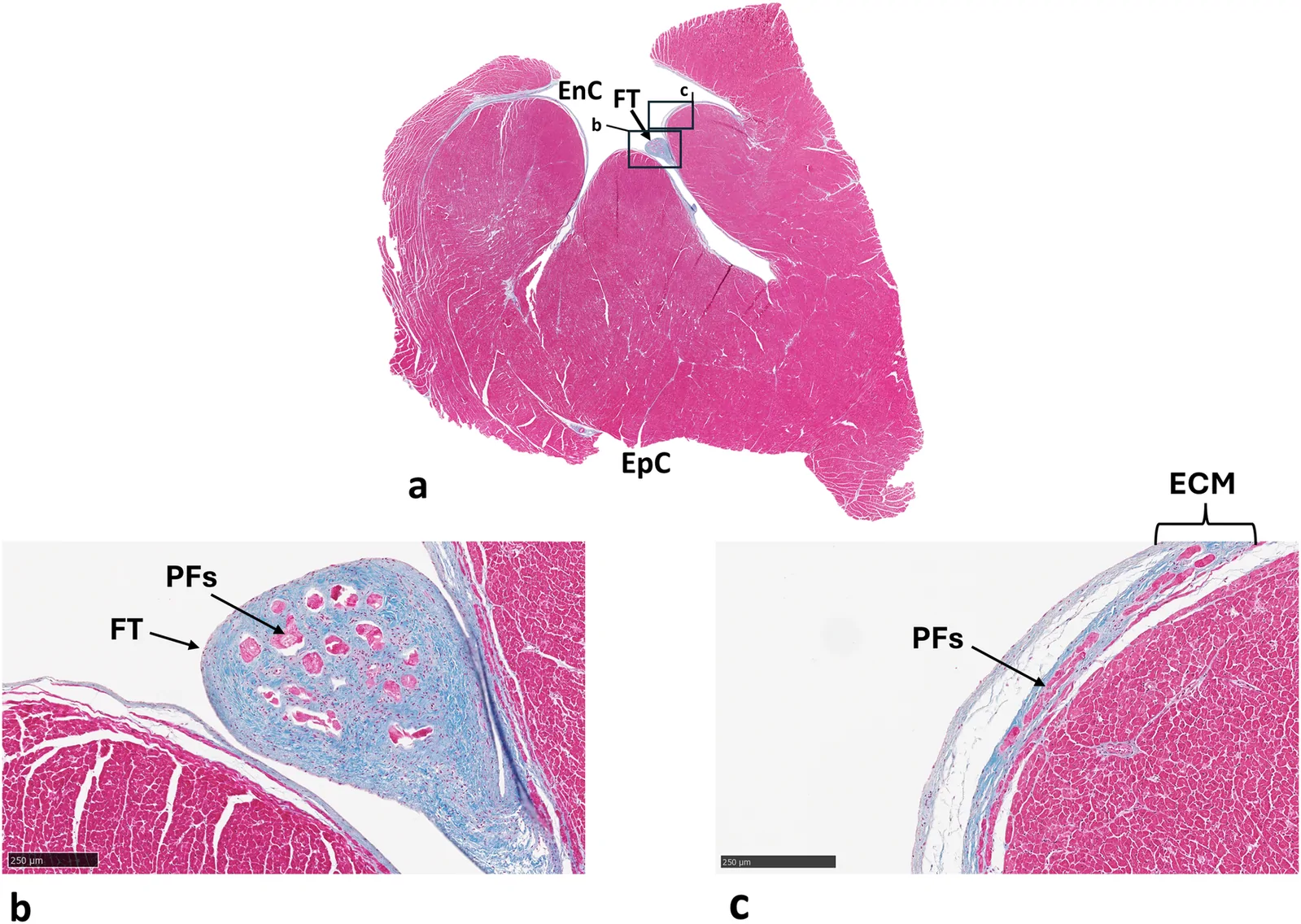
Masson’s trichrome-stained cross-section illustrating PF networks in the left ventricle (LV). **a** Low-magnification view of a transverse section from the LV apical region near the base of the papillary muscles, corresponding to the anatomical region highlighted in Fig. 1. Boxed areas indicate the locations of the enlarged views shown in **b** and **c**. **b** Transverse section of a free-running FT, showing a central PF bundle embedded within collagen-rich strands and surrounded by an endothelial layer. **c** Cross-section of the subendocardial connective tissue layer, showing PF bundles (pale red) interwoven with collagen-rich strands beneath the endocardium and superficial to the working myocardium. ECM, Extracellular matrix; EnC, Endocardium; EpC, Epicardium; LV, Left ventricle

Masson’s trichrome staining demonstrated the characteristic arrangement of PFs within both the subendocardial connective tissue layer and free-running FTs (Fig. 3). In the subendocardial region, PFs appeared as pale red myocytes embedded within collagen-rich strands beneath the endocardium. Within the FTs, PF bundles were similarly identified but enclosed by a denser collagen sheath and lined by an endothelial layer. These histological features define the structural reference used for OD-based tissue differentiation and subsequent interpretation of MRI relaxation parameters.

To assess section-to-section reproducibility of OD measurements, five serial sections from one representative left ventricular sample were analyzed (Supplementary Fig. S1). OD values for myocardium, PFs, and collagen showed minimal variability across sections. Myocardial OD_R and OD_B were highly consistent (0.07 ± 0.01 and 0.23 ± 0.01, respectively), as were collagen OD_R and OD_B (0.11 ± 0.01 and 0.08 ± 0.01). PFs exhibited slightly wider OD ranges (OD_R: 0.07 ± 0.02; OD_B: 0.17 ± 0.02), consistent with their mixed cellular and extracellular composition. Overall, the narrow inter-section variation across tissue classes supports the stability of staining and segmentation procedures and justifies pooling of sections for quantitative analyses.

**Fig. 4:**
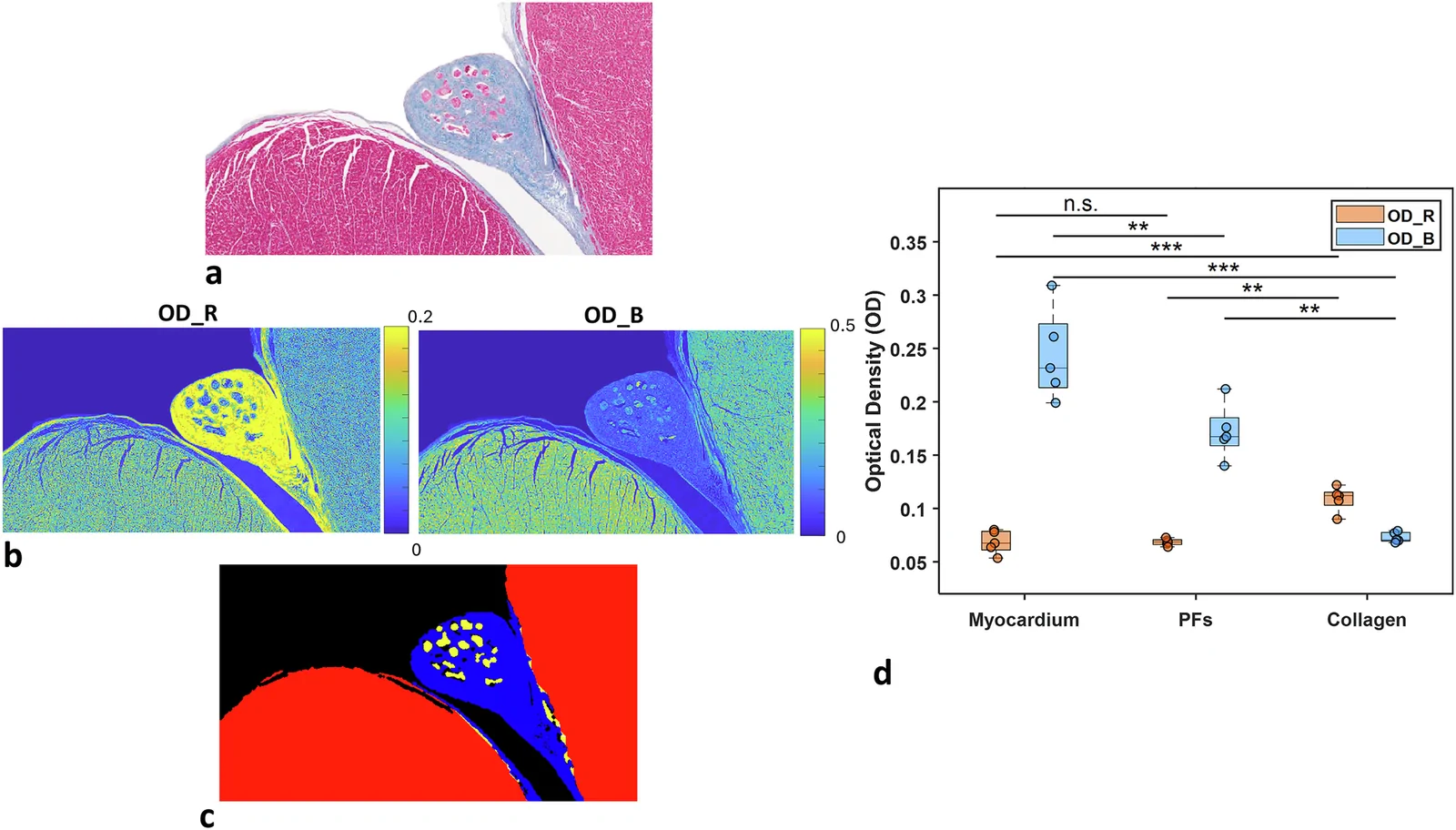
Histology-based tissue segmentation and OD comparison. **a** Example Masson’s trichrome-stained section containing an FT, showing myocardium, collagen, and PFs. **b** Corresponding OD maps obtained from red (OD_R) and blue channel (OD_B) decomposition, illustrating distinct staining profiles across tissue types. **c** Final segmentation mask classifying myocardium, PFs, and collagen using threshold-based separation of OD_R and OD_B. These segmented regions were used for quantitative analysis. **d** Boxplots of mean OD_R and OD_B for myocardium, PFs, and collagen. Measurements were obtained from two histological sections in five cardiac samples (*N* = 10). Statistical comparisons were performed using independent *t*-tests with Benjamini–Hochberg correction. Significance levels: n.s., *p* > 0.05; ^*^p < 0.05; ^**^p < 0.01; ^***^p < 0.001. ECM, Extracellular matrix

**Fig. 5:**
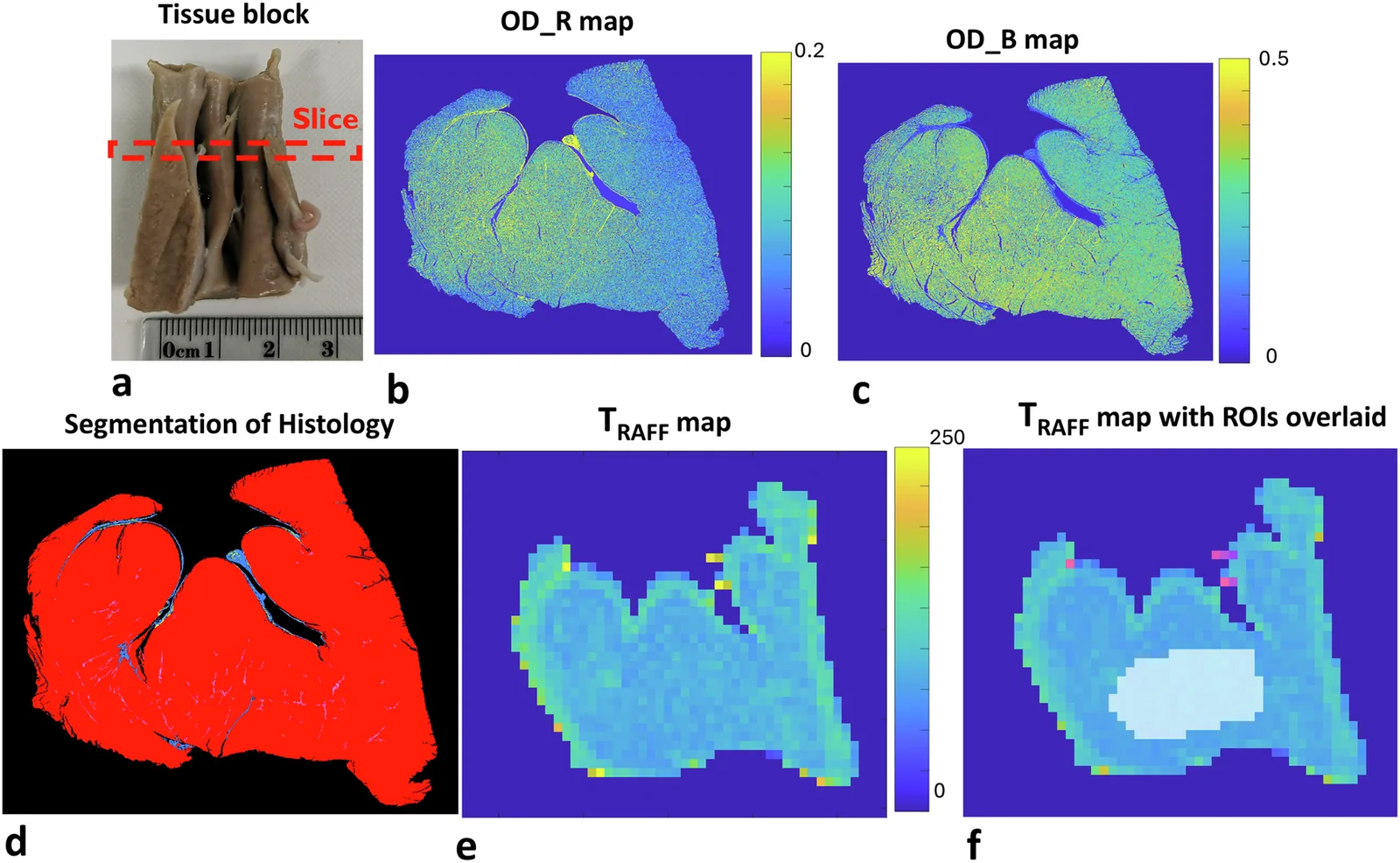
**a** Photograph of the tissue block used for the histological analyses shown in Figs. 2–4. **b**, **c** OD maps obtained from red (OD_R) and blue channel (OD_B) decomposition, illustrating distinct staining profiles across tissue types. **d** Final segmentation mask classifying myocardium, PFs, and collagen using threshold-based separation of OD_R and OD_B. **e** Corresponding RAFF relaxation time map. **f** RAFF relaxation time map overlaid with histology-derived ROIs, showing myocardium (white shaded area) and high-collagen regions (magenta shaded area)

The Masson’s trichrome-stained section illustrates myocardium, collagen-rich regions, and PF-associated structures within a FT (Fig. 4a). OD maps derived from the red and blue channels demonstrated distinct staining characteristics among the analyzed tissue regions (Fig. 4b). Threshold-based segmentation delineated the three tissue classes (Fig. 4c). Boxplots of mean OD_R and OD_B values showed that collagen-rich regions exhibited the highest OD_R (0.11 ± 0.01) and the lowest OD_B (0.07 ± 0.01), whereas myocardium showed lower OD_R (0.07 ± 0.01) and the highest OD_B (0.24 ± 0.04). PF-associated regions demonstrated OD_R values comparable to myocardium (0.07 ± 0.01, *p* = 0.99), while OD_B values were significantly lower than myocardium (0.17 ± 0.03, *p* < 0.01). In contrast, myocardium and collagen-rich regions, as well as PF-associated regions and collagen-rich regions, differed significantly in both OD_R and OD_B (*p* < 0.01 for all comparisons). Detailed statistical comparisons, including effect sizes and confidence intervals, are provided in Supplementary Table S1. These findings indicate that differentiation of PF-associated regions was primarily driven by the surrounding collagen-rich ECM environment rather than intrinsic myocardial staining differences. A photograph of the tissue block used for the histological analyses shown in Figs. 2 and 3 is presented (Fig. 5a). OD maps derived from red and blue channels illustrate spatially distinct staining patterns (Fig. 5b, c). Threshold-based segmentation provides a quantitative structural reference (Fig. 5d). The corresponding RAFF2 relaxation time map demonstrates regional variations in areas corresponding to histologically defined collagen-rich PF regions (Fig. 5e).

**Fig. 6:**
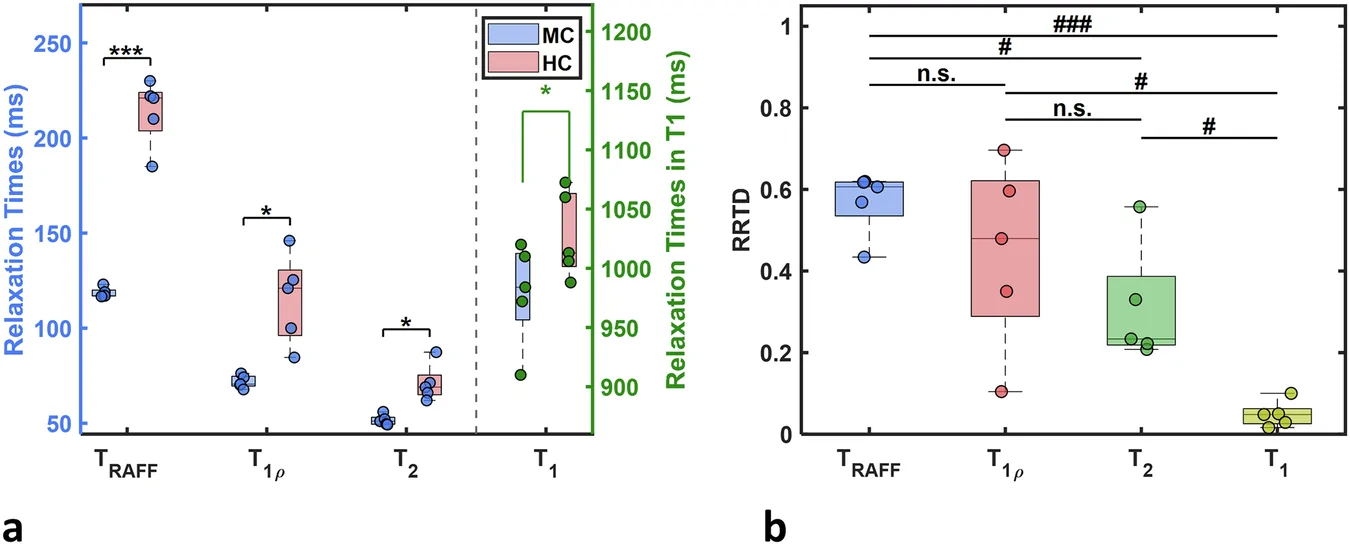
**a** Relaxation times measured from different qMRI parameters between myocardium control (MC) and high-collagen (HC) regions. **b** RRTD between HC and remote myocardium for each qMRI parameter. Statistical significance from pairwise comparison is indicated as  ^*^p < 0.05, ^**^p < 0.01 and ^***^p < 0.001 for differences in relaxation times between HC and myocardium, and as ^#^*p* < 0.05, ^##^*p* < 0.01, and ^###^*p* < 0.001 for differences in RRTD among qMRI parameters. n.s., not significant

Across five cardiac samples, qMRI revealed consistent relaxation-time differences between HC and MC regions (Fig. 6a). HC regions demonstrated markedly increased *T*_RAFF_ (214 ± 18 *versus* 119 ± 3 ms, *p* < 0.001), *T*_1ρ_ (115 ± 24 *versus* 72 ± 3 ms, *p* < 0.05), and *T*_2_ relaxation times (71 ± 10 *versus* 52 ± 3 ms, *p* < 0.05) compared with MC. *T*_1_ values were also higher in HC than MC regions (1028 ± 36 *versus* 979 ± 43 ms, *p* < 0.05), although the relative contrast remained lower than that observed for rotating-frame relaxation parameters. Detailed statistical comparisons, including effect sizes and confidence intervals, are provided in Supplementary Table S2.

The contrast between HC and MC regions, quantified using RRTD, varied among the qMRI parameters (Fig. 6b). *T*_RAFF_ demonstrated the highest RRTD (0.57 ± 0.08), followed by *T*_1ρ_ (0.45 ± 0.23) and *T*_2_ (0.31 ± 0.15), whereas *T*_1_ exhibited the lowest relative contrast (0.05 ± 0.03). Pairwise comparisons showed that *T*_RAFF_ RRTD was significantly greater than *T*_2_ (mean difference = 0.26, 95% CI: 0.09–0.42, *p* < 0.05) and *T*_1_ (mean difference = 0.52, 95% CI: 0.45–0.60, *p* < 0.001), with no significant difference between *T*_RAFF_ and *T*_1ρ_ (mean difference = 0.12, 95% CI: -0.17 to 0.42, *p* = 0.31). *T*_1ρ_ and *T*_2_ also demonstrated significantly higher RRTD values than *T*_1_ (mean difference = 0.40, 95% CI: 0.12–0.68, *p* < 0.05; and mean difference = 0.26, 95% CI: 0.12–0.41, *p* < 0.05, respectively), whereas the difference between *T*_1ρ_ and T_2_ did not reach statistical significance (mean difference = 0.14, 95% CI: −0.19 to 0.46, *p* = 0.31).

## Discussion

This study used histology as a structural reference to assess the sensitivity of clinically compatible qMRI techniques to collagen-rich PF-associated ECM pathways in the porcine left ventricle. Histological analysis combined with OD mapping demonstrated reproducible differentiation of myocardium, PF-associated regions, and collagen-rich tissue based on their distinct staining characteristics. These findings provide a consistent structural framework for interpreting the observed qMRI contrast and support the concept that relaxation-based MRI contrast in PF-associated regions primarily reflects the surrounding ECM rather than direct imaging of PF structures themselves [18].

Masson’s trichrome staining was selected because it provides robust and reproducible contrast between collagen and myocardium, making it particularly well suited for segmentation and for quantifying collagen-rich substrates associated with the CCS. This property is critical when using histology as a structural reference for correlating tissue microstructure with MRI-based relaxation parameters. Although other stains, such as Periodic acid-Schiff (PAS) and Movat’s pentachrome, have been employed in prior conduction system studies, Masson’s trichrome was applied consistently throughout this work to ensure standardized comparison across samples and to support reliable histology-referenced interpretation of the MRI findings [3, 7].

Establishing this histological reference is essential for clarifying the origin of MRI contrast in the context of the CCS. Using histology as a structural reference to interpret qMRI findings, this study indicates that MRI sensitivity to PF-associated regions arises not from direct visualization of the fibers themselves, but from detecting their co-localized, collagen-rich ECM within FTs and subendocardial layers. This distinction is critical for interpreting relaxation-time contrast and highlights the role of ECM pathways as the primary MRI-visible substrates associated with PFs. Consistent with this interpretation, PF-associated regions demonstrated OD_R values comparable to myocardium, whereas significant differences were observed in OD_B, indicating that differentiation was primarily driven by the surrounding collagen-rich ECM environment rather than intrinsic myocardial staining differences within PF structures themselves.

qMRI revealed distinct relaxation-time signatures in collagen-rich PF-associated regions, with *T*_RAFF_ and *T*_1ρ_ showing the largest relative increases compared with remote myocardium, followed by *T*_2_, while *T*_1_ exhibited lower relative contrast compared with the other evaluated parameters. This hierarchy may reflect the differing sensitivities of these parameters to tissue microstructure and is consistent with our previous rotating-frame relaxation studies of the CCS, in which *T*_RAFF_ and *T*_1ρ_ showed greater sensitivity to collagen-rich and microstructurally distinct conduction tissues than conventional *T*_1_ and *T*_2_ mapping at clinically feasible field strengths [13, 14]. *T*_RAFF_ also demonstrated the largest and most consistent relaxation differences across samples, which may suggest increased sensitivity to conduction-related microstructural differences. However, given the limited sample size and exploratory nature of the present study, these findings should be interpreted cautiously and require confirmation in larger studies. Rotating-frame relaxation methods such as *T*_RAFF_ and *T*_1ρ_ are particularly sensitive to low-frequency molecular motions, chemical exchange processes, and macromolecular environments, making them well suited for detecting collagen-rich ECM and bound water compartments associated with Purkinje fiber substrates. In contrast, *T*_2_ is influenced by tissue heterogeneity and water mobility, whereas *T*_1_ predominantly reflects bulk water content, which may explain its lower relative sensitivity in this context.

Alternative imaging approaches, including contrast-enhanced micro-computed tomography, have demonstrated the ability to visualize the Purkinje network and collagenous conduction pathways at high spatial resolution in *ex vivo* preparations [19, 20]. However, these techniques typically require specialized contrast agents, ionizing radiation, and are limited to post-mortem or experimental settings. In comparison, the MRI approach presented here leverages sequences and spatial resolutions achievable on clinical 3-T scanners, offering a non-invasive and potentially translatable means of probing PF-associated collagen substrates indirectly through their relaxation properties. Together, these findings suggest the feasibility of MRI-based tissue characterization for studying conduction-related microstructure beyond the spatial resolution limits of conventional imaging.

Although this study focused on PFs, other components of the CCS, including the sinoatrial node (SAN) and atrioventricular conduction axis (AVCA), also play a critical role in arrhythmias and conduction disorders [21]. Rotating-frame relaxation mapping techniques such as *T*_RAFF_ and *T*_1ρ_ show broader potential for characterizing conduction-related microstructure beyond PFs. Extending beyond the present findings, our previous studies demonstrated that rotating-frame relaxation methods enable contrast-agent–free visualization of the SAN and AVCA in *ex vivo* swine hearts, reflecting their distinct cellular organization and collagen-rich environments [13, 14]. These observations suggest that the sensitivity of rotating-frame relaxation to macromolecular content and microstructural organization represents a common contrast mechanism across multiple elements of the CCS.

Despite these advances, several limitations should be considered. First, the sample size was modest, reflecting the exploratory feasibility nature of this *ex vivo* study. This was partly due to the technically demanding and time-intensive workflow, including multi-sequence MRI acquisition, histological processing, and slice-level anatomical correspondence between MRI and histology. Although ten sections were analyzed, these were derived from five hearts, limiting the number of independent biological samples available for statistical analysis. Second, histological and OD analyses are inherently destructive and limited to *ex vivo* validation. Tissue deformation during histological processing, imperfect anatomical correspondence between histology and MRI slices, and partial-volume effects may have influenced measurements in thin and heterogeneous PF-associated regions. Third, although *T*_RAFF_ and *T*_1ρ_ showed increased sensitivity to collagen-rich PF-associated ECM pathways, these relaxation changes are not specific to PF-associated collagen and may also occur in other collagen-rich myocardial conditions, such as fibrosis or post-infarction scar tissue. Finally, all MRI data were acquired *ex vivo*, and no dedicated assessment of B0/B1-related artifacts was performed in the present setup. In addition, the spatial resolution currently achievable with clinical *in vivo* cardiac MRI remains insufficient for direct visualization of individual PF structures. Therefore, future *in vivo* applications would likely rely on detecting PF-associated collagen-rich substrates rather than resolving microscopic PF architecture directly. Further optimization will also be required to address cardiac motion, B0/B1 inhomogeneity, SAR constraints, and practical acquisition limitations.

Building on the histology-referenced framework established, translation of this approach toward *in vivo* imaging remains challenging and was beyond the scope of the present study. Future development will likely require improved spatial resolution, efficient acquisition strategies, and integration with anatomical landmarks or complementary structural MRI techniques to improve localization of PF-associated ECM pathways under clinical conditions. Importantly, future *in vivo* applications would likely rely on detecting PF-associated collagen-rich substrates rather than direct visualization of individual PF structures, which remain below the spatial resolution limits of current clinical MRI systems. In addition, larger studies across species, disease models, and pathological substrates will be necessary to evaluate the robustness and generalizability of the observed histology–MRI relationships. Collectively, these efforts may support future investigation of conduction-related microstructure and ventricular conduction abnormalities using non-invasive MRI approaches.

## Conclusion

Using OD-based histology as a structural reference, this study demonstrates that qMRI relaxation times are sensitive to collagen-rich PF-associated pathways in the *ex vivo* porcine heart. OD mapping enabled consistent differentiation of myocardium, collagen-rich tissue, and PF-associated regions, while qMRI revealed elevated *T*_RAFF_, *T*_1ρ_, *T*_2_, and *T*_1_ values in collagen-rich PF-associated regions, with *T*_RAFF_ and *T*_1ρ_ providing the strongest relative contrast. The observed agreement between histological tissue characteristics and MRI relaxation behavior supports the interpretation that these relaxation-based imaging techniques are sensitive to PF-associated collagen-rich ECM substrates. These findings support further investigation of non-invasive MRI approaches for characterizing conduction-related cardiac microstructure and ventricular conduction substrates.

## Supplementary information

**Additional File 1: Fig. S1.** Boxplots showing the distribution of mean optical density (OD_R and OD_B) in myocardium, Purkinje fibers (PFs), and collagen across five consecutive sections from one representative cardiac sample. Each point represents one section. The results show minimal section-to-section variation, confirming the reproducibility of manual staining and image quantification procedures. No statistical comparisons were made, as the purpose was to verify measurement consistency. **Table S1.** Statistical comparisons of histological optical density measurements among myocardium, PF-associated regions, and collagen. **Table S2.** Statistical comparisons of qMRI parameters between MC and HC regions.

## Data Availability

The datasets generated and analyzed during the current study are available from the corresponding author upon reasonable request.

## Abbreviations

CCS: Cardiac conduction system
ECM: Extracellular matrix
FT: False tendon
OD: Optical density
PFs: Purkinje fibers
qMRI: Quantitative MRI
ROI: Region of interest
RRTD: Relative relaxation time difference
RAFF: Relaxation along the fictitious field
