# Supplementary material for: Histology-validated quantitative MRI characterization of collagen-rich Purkinje fiber-associated pathways in the ex vivo porcine heart
Source: Eur Radiol Exp. 2026 Sep 15;10:132. doi: 10.1186/s41747-026-00793-0 (PMC13578148; doi:10.1186/s41747-026-00793-0)
Supplement: Supplementary file 1 [file 41747_2026_793_MOESM1_ESM.pdf]

Histology-validated quantitative MRI characterization of collagen-rich Purkinje fiber-associated pathways in the ex vivo porcine heart

ELECTRONIC SUPPLEMENTARY MATERIAL

Fig. S1

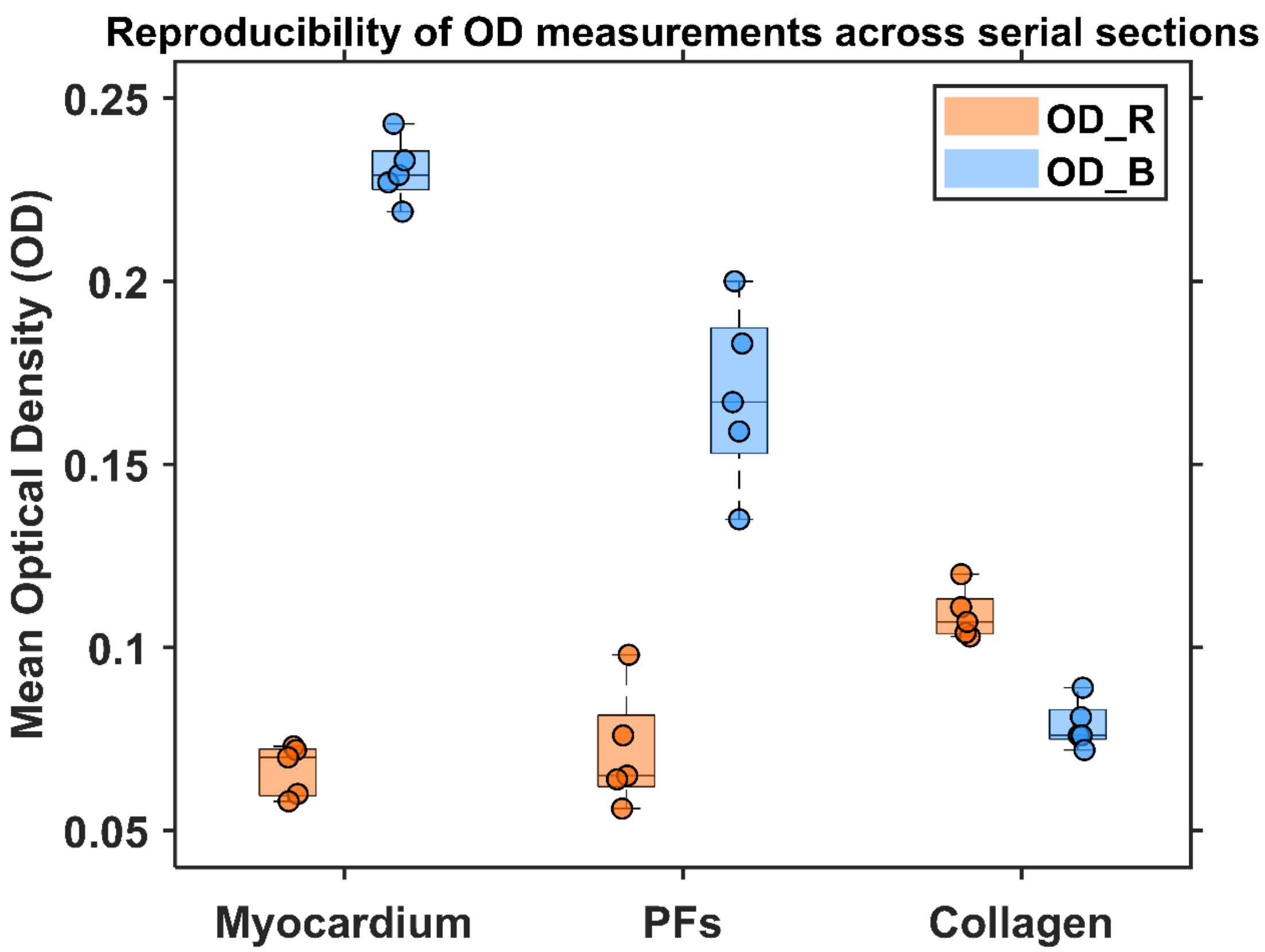

Fig. S1 Boxplots showing the distribution of mean optical density (OD_R and OD_B) in myocardium, Purkinje fibers (PFs), and collagen across five consecutive sections from one representative cardiac sample. Each point represents one section. The results show minimal section-to-section variation, confirming the reproducibility of manual staining and image quantification procedures. No statistical comparisons were made, as the purpose was to verify measurement consistency.

Eur Radiol Exp (2026) Li Y; Casula V; Pradeep M; Liimatainen T

Table S1. Statistical comparisons of histological optical density measurements among myocardium, PF-associated regions, and collagen.

Comparison	Tissue 1 (mean ± SD)	Tissue 2 (mean ± SD)	Mean difference (Tissue 2 −Tissue 1)	95 % CI	P value	Cohen's d_z
Myocardium vs PF (OD_R)	0.07 ± 0.01	0.07 ± 0.003	-0.0001	-0.0156 to 0.0154	0.98	-0.008
Myocardium vs PF (OD_R)	0.24 ± 0.04	0.17 ± 0.03	-0.07	-0.1016 to -0.0417	0.003	-2.97
Myocardium vs Collagen (OD_R)	0.69 ± 0.01	0.11 ± 0.01	0.04	0.0331 to 0.0476	0.0001	6.93
Myocardium vs Collagen (OD_B)	0.24 ± 0.04	0.07 ± 0.005	-0.17	-0.2238 to -0.1179	0.0008	-4
PF vs Collagen (OD_R)	0.07 ± 0.003	0.11 ± 0.011	0.04	0.0238 to 0.0570	0.0025	3.02
PF vs Collagen (OD_B)	0.17 ± 0.03	0.07 ± 0.005	-0.0992	-0.1330 to -0.0654	0.0012	-3.64

PF, Purkinje fiber-associated region; OD, optical density; CI, confidence interval. Mean differences were calculated as Tissue 2 − Tissue 1.

Table S2. Statistical comparisons of qMRI parameters between MC and HC regions.

Comparison	MC region (mean ± SD)	HC region (mean ± SD)	Mean difference (HC − MC)	95 % CI	P value	Cohen's d_z
T_{RAFF2}	119 ± 2 ms	214 ± 18 ms	95 ms	74 to 116 ms	0.00024	5.55
$T_{1\rho}$	72 ± 3 ms	115 ± 24 ms	44 ms	12 to 76 ms	0.019	1.69
T_2	52 ± 3 ms	71 ± 10 ms	20 ms	6 to 33 ms	0.016	1.80
T_1	979 ± 43 ms	1028 ± 36 ms	49 ms	11 to 87 ms	0.023	1.60

MC, remote myocardium region; HC, collagen-rich region; CI, confidence interval. Mean differences were calculated as HC − MC.
